# NUP37, a positive regulator of YAP/TEAD signaling, promotes the progression of hepatocellular carcinoma

**DOI:** 10.18632/oncotarget.20336

**Published:** 2017-08-18

**Authors:** Xiaoling Luo, Yuting Liu, Weiguang Feng, Liu Lei, Yemu Du, Jinsheng Wu, Shaochuang Wang

**Affiliations:** ^1^ Department of Gastroenterology, Huai’an First People's Hospital, Nanjing Medical University, Huai’an, Jiangsu Province 223300, P. R. China; ^2^ The Fourth People's Hospital of Huai’an, Huai’an, Jiangsu Province 223002, P. R. China; ^3^ Department of Hepatobiliary & Pancreatic Surgery, Huai’an First People's Hospital, Nanjing Medical University, Huai’an, Jiangsu Province 223300, P. R. China

**Keywords:** NUP37, YAP/TEAD signaling, cell growth and motility, hepatocellular carcinoma

## Abstract

Activation of YAP/TEAD signaling is very common in the progression of HCC (Hepatocellular carcinoma). Nuclear pore complex (NPC) regulates the shuttling of proteins between cytoplasm and nucleus. Nuclear accumulation of YAP protein has been observed in the majority of HCC tissues. However, whether NPC could regulate the YAP/TEAD signaling remains unknown. In this study, it was found NUP37, the component of NPC, significantly up-regulated in HCC clinical samples and mouse model. Over-expression of NUP37 promoted the growth, migration and invasion of HCC cells, while knocking down the expression of NUP37 inhibited the growth, migration, invasion and metastasis of HCC cells and improved the survival of the mouse model. NUP37 interacted with YAP and activated YAP/TEAD signaling by enhancing the interaction between YAP and TEAD. Taken together, these data demonstrated the oncogenic roles of NUP37 in the progression of HCC and suggested that NUP37 might be a promising therapeutic target.

## INTRODUCTION

Hepatocellular carcinoma (HCC), a malignancy with poor survival, is prevalent in Asia contries, especially China. Aberrant activation of several signaling pathways, such as Wnt/beta-catenin signaling [1], Ras-ERK signaling and chromatin remodeling pathway [2, 3], have been reported to link to the tumorigensis of HCC. Recently, several studies have demonstrated that activation of YAP/TEAD signaling promoted the development of HCC [4, 5]. However, the molecular mechanisms leading to the activation of YAP/TEAD signaling are not fully understood.

YAP (Yes-associated protein) is the key component of Hippo signaling which controls the sizes of the organs [6]. Hippo kinases MST1/2 tightly control the location and protein level of YAP by promoting its phosphorylation and degradation [7]. It has been demonstrated that high density of the cell culture activated the kinase activity of MST1/2 and led to the cytoplasmic localization of YAP [6]. In cancer cells, due to loss function of upstream regulator, YAP was accumulated in the nucleus where it formed a complex with TEAD and activated a panel of growth-related and growth-relate genes, such as CYR61, CTGF and Cyclin E [6, 8] The shuttle of YAP from the cytoplasm to the nucleus through nuclear pore complex (NPC) is essential for its oncogenic roles. However, whether the NPC regulates YAP/TEAD signaling remains unknown.

The nucleus is enclosed with the nuclear envelope. The passage across the nuclear envelope is mediated by the nuclear pore complex (NPC). NPC is estimated to contain about 30 proteins including Nups and Nucleoporins [9]. Besides the well-known transport function, NPCs participate in a number of additional cellular processes, including nuclear organization, cell cycle regulation signaling transduction [9]. NUP37 is a component of NPC. Previous study has shown the NUP37 is both a significant mutated genes and a tumor-destructive genes in oral squamous cell carcinoma, suggesting that NUP37 might function in the tumorigenesis [10]. However, up to date, the expression pattern, the functions and the molecular mechanism for NUP37 in the tumorigenesis have not been reported.

In this study, we examined the expression of NUP37 in the HCC and investigated its biological functions as well as molecular mechanism.

## RESULTS

### The expression of NUP37 was elevated in HCC

**Figure 1:**
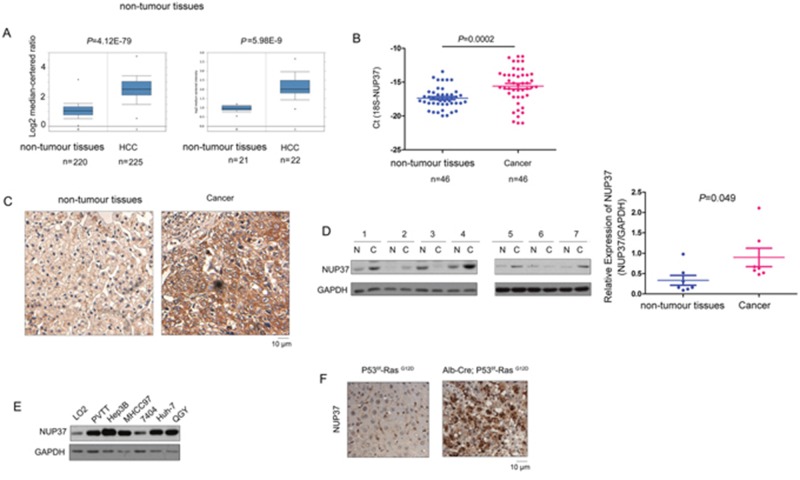
NUP37 was up-regulated in HCC clinical samples and mouse model **(A)** Mining the oncomine database revealed up-regulation of NUP37 in two independent cohorts. One cohort included 220 normal liver tissues and 225 HCC tissues. Another cohort contained 21 normal liver tissues, 22 HCC tissues. **(B)** NUP37 mRNA level was elevated in HCC tissues compared with the paired non-tumor tissues. The mRNA level of NUP37 was examined in 46 HCC tissues and the paired non-tumor tissues using Real-time PCR. The mRNA level of 18S was used as internal control. **(C)** IHC staining revealed the up-regulation of NUP37 in HCC tissues. **(D)** The protein level of NUP37 in HCC tissues and paired non-tumor tissues was examined using western blot analysis. The results were quantified. **(E)** The protein level of NUP37 in normal hepatic cells (LO2) and HCC cells (PVTT, 7404, Huh-7, Hep3B, QGY, MHCC97). **(F)** The protein level of NUP37 was elevated in the hepatic tissues of the HCC mouse model (Alb-Cre; P53^f/f^; Ras^G12D^).

To explore the expression of NUP37 in the HCC, we first turned to the Oncomine database to mine the expression pattern of NUP37 in HCC. Down-regulation of NUP37 was observed in two independent studies (Figure 1A). To confirm these findings, we examined the NUP37 mRNA level in 46 HCC samples and paired non-cancerous tissues. Consistent with the findings from Oncomine, up-regulation of NUP37 in HCC tissues was found, and the mRNA level of NUP37 was about five fold higher in HCC tissues (Figure 1B). In the next study, we examined the protein level of NUP37 in HCC using immunohistochemistry (IHC) and western blot analysis. Elevated NUP37 protein level was detected in HCC tissues both in the IHC study and western blot analysis (Figure 1C-1D). In the next study, the expression of NUP37 in a panel of HCC cell lines was assessed. Low expression of NUP37 was observed in the normal liver cell line (LO2) and high expression of NUP37 was observed in HCC cell line (PVTT, Hep3B, MHCC97, 7404, Huh-7 and QGY) (Figure 1E). Furthermore, up-regulation of NUP37 was found in the HCC mouse model driven by both loss of P53 and activation of Ras (Alb-Cre; P53^f/f^; Ras^G12D^) (Figure 1F). Taken together, these data suggested that NUP37 was up-regulated in the progression of HCC.

### NUP37 promoted the growth, migration and invasion of HCC cells

**Figure 2:**
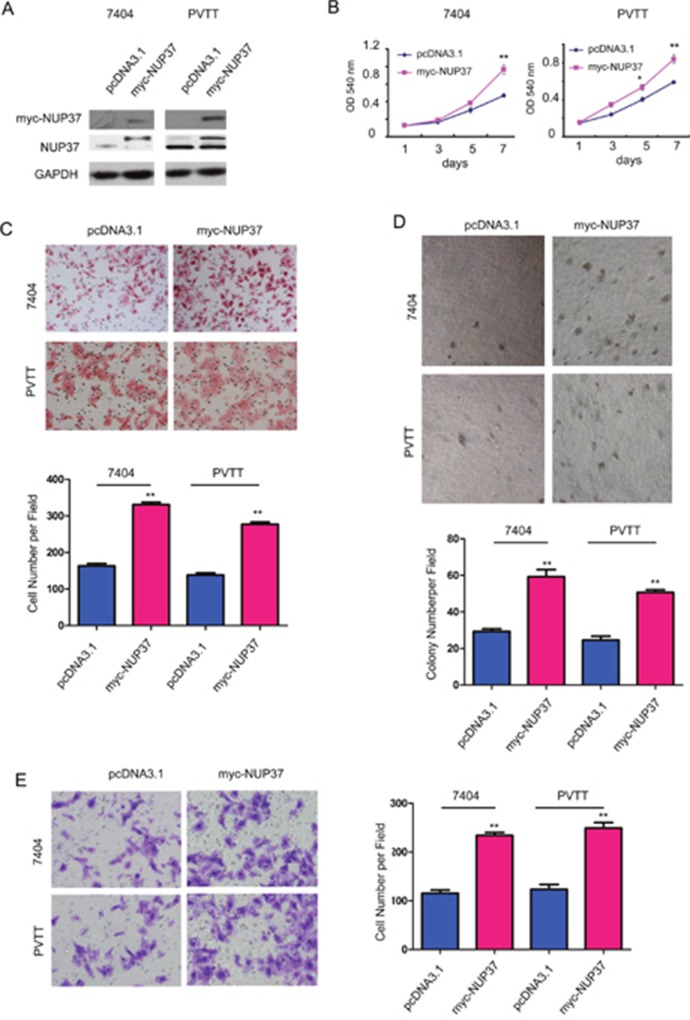
NUP37 positively regulated the growth, migration, colony formation and invasion of HCC cells **(A)** Ectopic expression of NUP37 in 7404 and PVTT cells. The exogenenously expressed NUP37 was examined using both anti-myc antibody and anti-NUP37 antibody. **(B)** MTT assay to evaluate the roles of NUP37 in the growth of 7404 and PVTT cells. **(C)** The Boyden chamber assay was used to evaluate the roles of NUP37 in the migration of 7404 and PVTT cells. **(D)** The soft agar assay was used to examine the function of NUP37 in the anchorage-independent growth of 7404 and PVTT cells. **(E)** The transwell was used to examine the roles of NUP37 in the invasion of 7404 and PVTT cells. ^*^, *P*<0.05; ^**^, *P*<0.01.

The up-regulation of NUP37 in HCC prompted us to study its roles in the progression of this malignancy. We first forced expression of NUP37 (myc-NUP37) in 7404 and PVTT cells (Figure 2A). The effects of myc-tagged NUP37 (myc-NUP37) on the growth, migration, invasion and colony formation of 7404 and PVTT cells were evaluated using MTT assay, Boyden chamber assay and soft agar assay. As shown in Figure 2, over-expression of NUP37 in 7404 and PVTT cells promoted the growth (Figure 2B), migration (Figure 2C), colony formation (Figure 2D) and invasion (Figure 2E) of cancer cells. These observations indicated the oncogenic roles of NUP37 in HCC cells.

**Figure 3:**
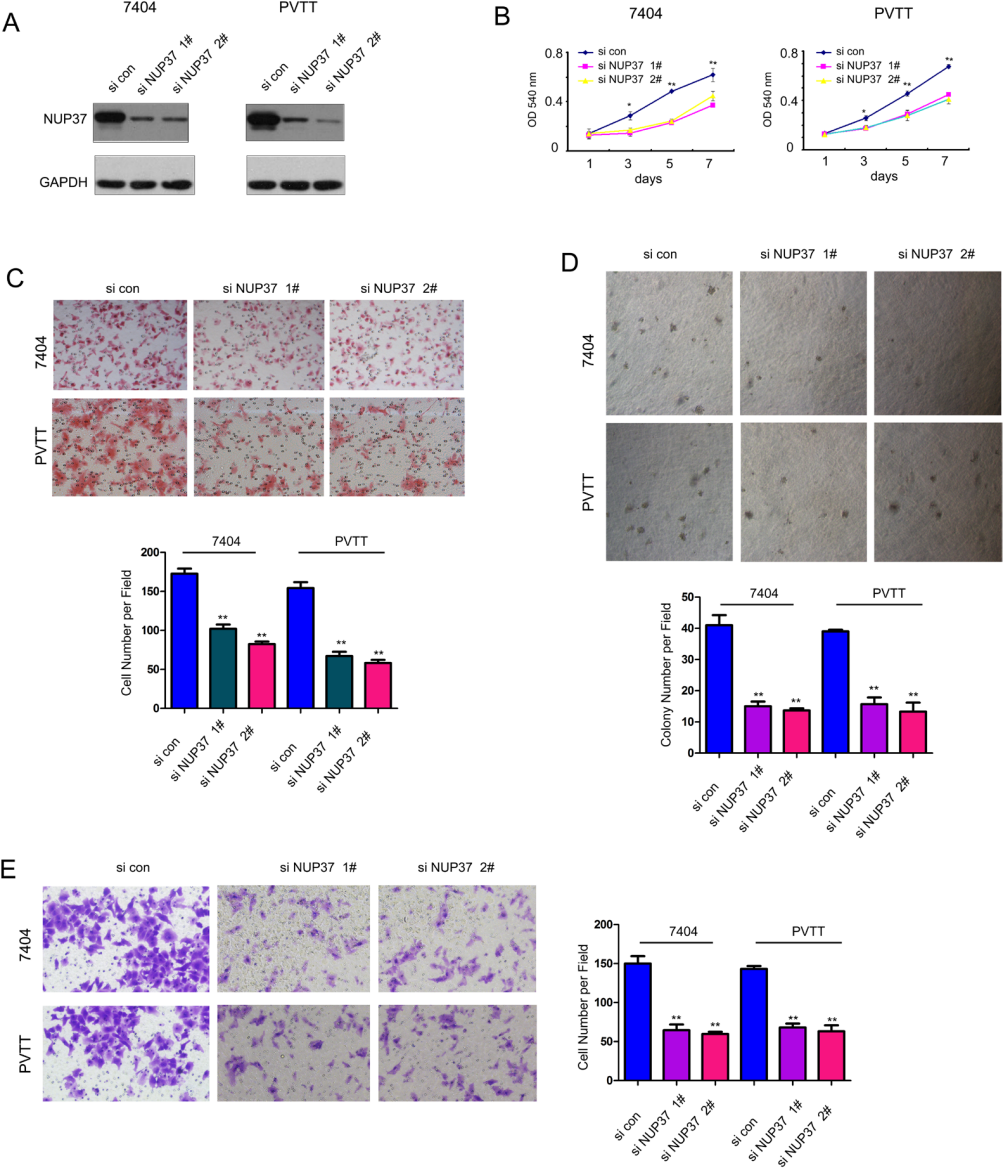
Down-regulation of NUP37 impaired the growth, migration, colony formation and invasion of HCC cells **(A)** Knocking down the expression of NUP37 in 7404 and PVTT cells using two independent si RNA sequences. **(B)** MTT assay to evaluate the effects of down-regulating NUP37 in the growth of 7404 and PVTT cells. **(C)** The Boyden chamber assay was used to evaluate the effects of down-regulating NUP37 in the migration of 7404 and PVTT cells. **(D)** The soft agar assay was used to examine the function of down-regulating NUP37 in the anchorage-independent growth of 7404 and PVTT cells. **(E)** The transwell was used to examine the effects of down-regulating NUP37 in the invasion of 7404 and PVTT cells. ^*^, *P*<0.05; ^**^, *P*<0.01.

Next, we evaluated the functions of endogenous NUP37 in 7404 and PVTT cells through knocking down the expression of NUP37 by two independent siRNA sequences (Figure 3A). Consistent with the findings shown in Figure 2, knocking down the expression of NUP37 inhibited the growth, migration, colony formation and invasion of the 7404 and PVTT cells (Figure 3B-3E). Collectively, these data suggested that NUP37 promoted the progression of HCC by enhancing the growth, migration, colony formation and invasion of cancer cells.

### NUP37 activated YAP/TEAD signaling in HCC cells

**Figure 4:**
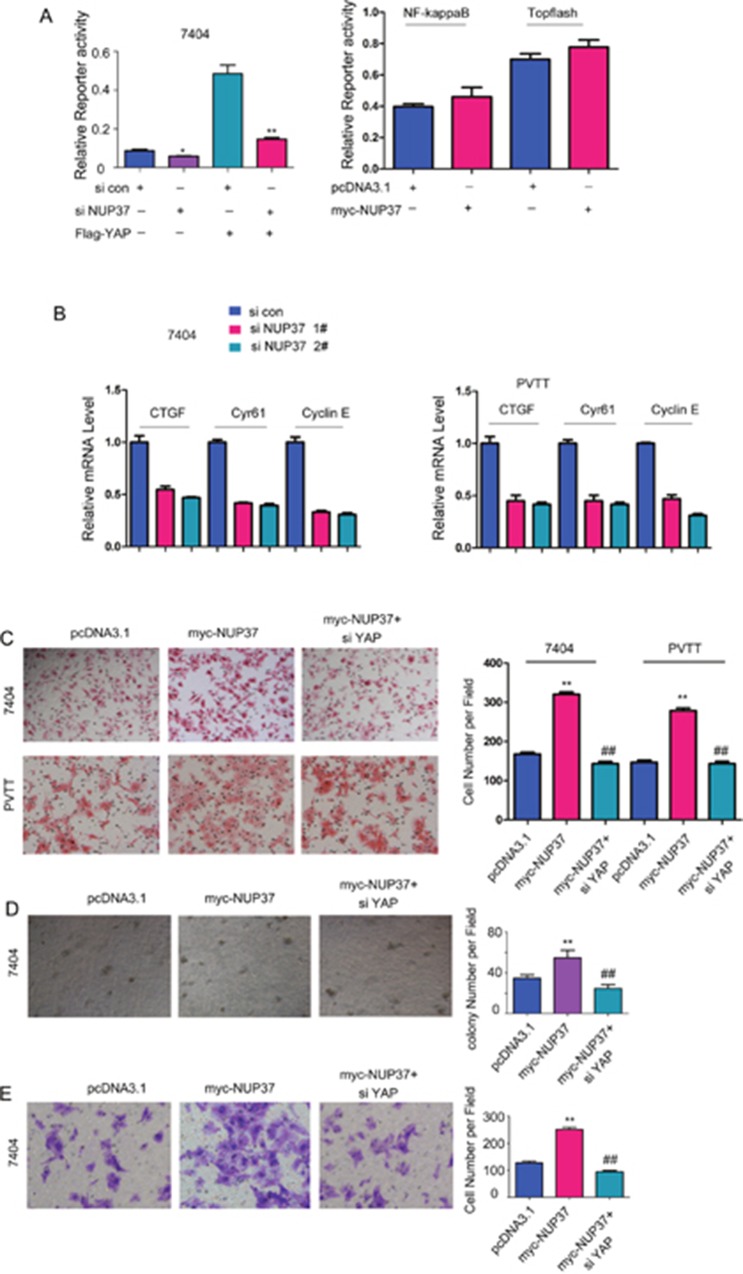
NUP37 positively activated YAP/TEAD signaling in HCC cells **(A)** Down-regulating NUP37 inhibited the reporter for the transcriptional activity of YAP/TEAD complex. However, down-regulating NUP37 exerted little effects on the reporter for NF-κB signaling and beta-catenin/TCF signaling (Topflash). **(B)** The target genes (CTGF, Cyr61 and Cyclin E) were down-regulated in cells being knocked down the expression of NUP37. **(C)** Knocking down the expression of YAP rescued the effects of NUP37 on the migration of 7404 and PVTT cells. **(D)** Knocking down the expression of YAP rescued the effects of NUP37 on the colony formation of 7404 cells. **(E)** Knocking down the expression of YAP rescued the effects of NUP37 on the invasion of 7404 cells.

To explore the underlying mechanisms through which NUP37 promoted the progression of HCC, we screened the effects of NUP37 on the activation of various signal pathways. As shown in Figure 4A, knocking down the expression of NUP37 inhibited the reporter activity driven by YAP/TEAD complex both at the basal level and upon the stimulation of exogenously expressed YAP while exerted little effects on the activity of reporter driven by NF-κB signaling and beta-catenin/TCF signaling (Topflash) (Figure 4A). In addition, knocking down the expression of NUP37 decreased the expression of several target genes (Cyr61, CTGF and cyclin E) downstream YAP/TEAD complex (Figure 4B). Furthermore, knocking down the expression of YAP rescued the effects of NUP37 on the migration, colony formation and invasion of HCC cells (Figure 4C-4E), suggesting the essential roles of NUP37 in the activation of YAP/TEAD signaling. In summary, these observations suggested that NUP37 promoted the growth, migration, colony formation and invasion of HCC cells possibly by activating the transcriptional activity of YAP/TEAD complex.

### NUP37 interacted with YAP in HCC cells

**Figure 5:**
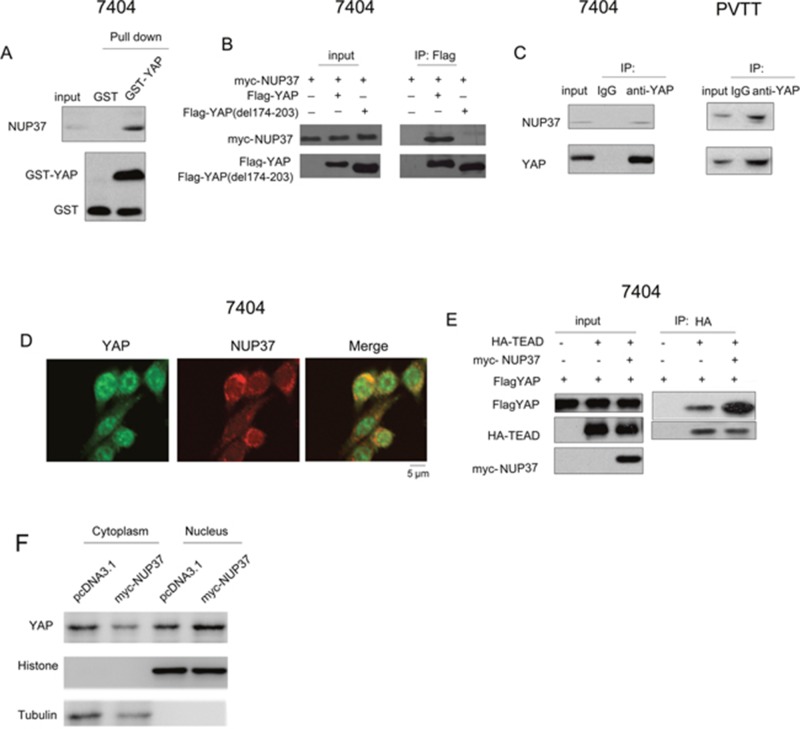
NUP37 formed a complex with YAP **(A)** The interaction between GST-YAP and NUP37 was examined using GST pull-down assay. GST pull-down assay was performed using 7404 cell lysate and GST-YAP fusion protein. **(B)** Ectopically expressed YAP (Flag-YAP), YAP mutant (del 174-203aa) and NUP37 (myc-NUP37) interacted in 7404 cells in the immunoprecipitation assay. **(C)** Endogenously expressed YAP and NUP37 formed a complex in 7404 and PVTT cells. **(D)** The fluorescence staining to examine the co-localization of NUP37 and YAP. **(E)** NUP37 enhanced the interaction between YAP and TEAD in the immunoprecipitation assay. **(F)** The nucleus-localized YAP protein in NUP37 overexpressing 7404 cells.

To understand the detail mechanism through which NUP activated the transcriptional activity of YAP/TEAD complex, we first tested the interaction between NUP37 and the components of YAP/TEAD complex. As shown in Figure 5A, the fusion protein GST-YAP interacted with the endogenous NUP37 (Figure 5A). In addition, exogenously expressed YAP (Flag-YAP) and NUP37 (myc-NUP37) formed a complex in the immunoprecipitation assay (Figure 5B). Moreover, the endogenously expressed NUP37 and YAP interacted with each other in 7404 and PVTT cells (Figure 5C). On the other hand, the fluorescence staining revealed the co-localization of NUP37 and YAP (Figure 5D). These data suggested that NUP37 formed a complex under the physiological conditions. Next, we tested the influence of NUP37expression on the interaction between YAP and TEAD. It was found that overexpression of NUP37 enhanced the interaction between YAP and TEAD (Figure 5E). Consistently, the level of nucleus-localized YAP protein was higher in 7404 cells overexpressing NUP37. At last, we screened the microdomain in the YAP for the binding of NUP37. It was found that deletion of 174-203aa of YAP abolished its interaction with NUP37 (Figure 5B). In summary, these observations suggested that NUP37 activated YAP/TEAD signaling by enhancing the interaction between YAP and TEAD.

### Down-regulating the expression of NUP37 suppressed the intra-hepatic metastasis

**Figure 6:**
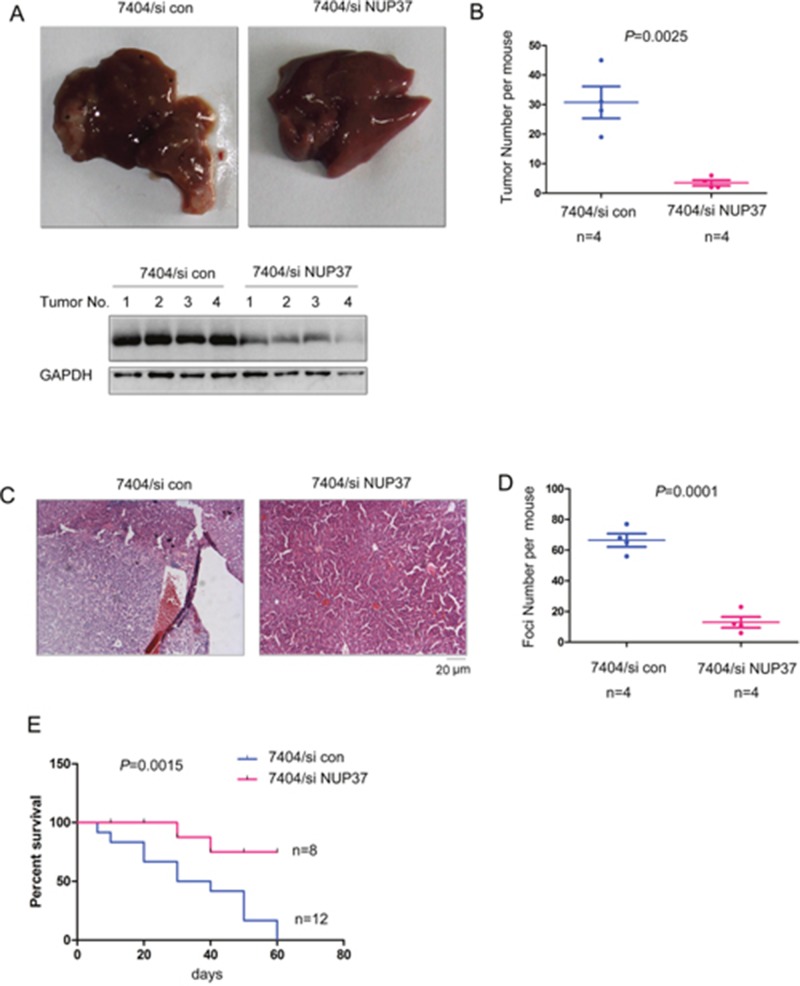
Knocking down the expression of NUP37 inhibited the metastasis of HCC **(A-B)** The intrahepatic metastasis was performed to examine the roles of NUP37 in the metastasis of HCC cells (A). The morphology of livers were shown and the tumor number was quantified (B). Note: Down-regulation of NUP37 was found in tumors formed by 7404/siNUP37 cells. **(C)** HE staining of the tumors shown in (A-B). **(D)** Quantification of metastatic foci in (C). **(E)** Survival curve of the mice in the intrahepatic metastasis assay.

Intrahepatic metastasis is very common in the progression of HCC. To evaluate whether NUP37 promoted the intrahepatic metastasis *in vivo*, we turned to the intrahepatic metastasis mouse model. 7404 cells were injected into the liver lobe. As the gross morphology shown in Figure 6A, injection of 7404 cells into one of the liver lobes led to significant intrahepatic metastasis. However, knocking down the expression of NUP37 inhibited the tumor formation in other lobes (Figure 6A-6B). Next, we performed the HE staining to examine the tumor foci (Figure 6C). As shown in Figure 6D, down-regulation of NUP37 repressed the metastatic foci formation. These data suggested that NUP37 is essential for the intrahepatic metastasis. Moreover, knocking down the expression of NUP37 in 7404 cells improved the survival of nude mice in the intrahepatic metastasis assay (Figure 6E). Collectively, these findings suggested that NUP37 promoted the metastasis of HCC.

## DISCUSSION

Several studies have demonstrated that conditioned knocking out Mst1/2 and Sav in the mice led to the accumulation of YAP in the nucleus, activation of YAP/TEAD signaling and the tumorigenesis of HCC [4, 11]. In the HCC clinical samples, nuclear accumulation of YAP was observed in most of the tumor tissues [12]. Nuclear pore complex (NPC) regulated the shuttle of YAP from the cytoplasm to the nucleus. However, whether NPC regulated the YAP/TEAD signaling in HCC remains unknown. This study showed that NUP37, a component of NPC, was up-regulated in both clinical HCC samples and mouse model. Functional analysis demonstrated that NUP37 promoted the growth, migration, invasion and metastasis of HCC cells possibly by activating YAP/TEAD signaling. Activation of YAP/TEAD signaling by NUP37 promoted the expression of CTGF, Cyr61 and Cyclin E, which has been reported as positive regulators for cell motility and growth.

How NUP37 activated YAP/TEAD signaling was further investigated. NUP37 was found to interact with YAP and enhanced the interaction between YAP and TEAD. Based on these findings, we speculated that NUP37 might enhance the import and/or the retention of YAP in the nucleus and thus promoted the interaction between YAP and TEAD. Similarly, NPC has been reported to be involved in the regulation of beta-catenin/TCF signaling [13, 14]. Recent evidence suggested that the primary nuclear transport route of beta-catenin was independent of the classical Ran/importin import machinery, and that beta-catenin directly contacted the nuclear pore complex to self-regulate its own entry into the nucleus. For example, NP62 has been reported to activate Wnt/beta-catenin by facilitating the nuclear import of beta-catenin [13, 14]. Moreover, the beta-catenin nuclear import pathway might provide an opportunity for identification of specific drug targets and inhibition of beta-catenin nuclear function [15]. These previous studies might provide a good explanation for the regulation of YAP signaling by NUP37.

Another important finding of this study is the induction of NUP37 in the HCC mouse model (Alb-Cre; P53^f/f^; Ras^G12D^), suggesting NUP37 might be a novel downstream effector of oncogenic Ras signaling. Oncogenic Ras signaling has been reported to activate YAP signaling by inducing the heterodimerization of Mst1 and Mst2 [6]. Our study indicated that Ras might activate YAP signaling by inducing the expression of NUP37, suggesting that Ras might activated YAP/TEAD signaling through different mechanisms.

In summary, this study demonstrated the oncogenic roles of NUP37 in the HCC, suggesting that NUP37 might be a promising therapeutic target.

## MATERIALS AND METHODS

### Tissue samples and cell cultures

The HCC tissues used in this study was collected from Huai’an First People's Hospital after the informed consent from the patients. Tissues were kept in −80°C. The RNA was extracted from HCC tissues using Trizol. Human normal liver cell line LO2 and HCC cell lines (7404, PVTT, MHCC97, Hep3B, QGY and Huh-7) were purchased from cell bank (the Chinese Academy of Science, Shanghai) and were cultured in DMEM medium (Gibco) supplemented with 10% (v/v) heat-inactivated FBS (Gibco-BRL) and 1% penicillin/streptomycin (Gibco-BRL). The cells were cultured at 37°C in a 5%CO2 humidified atmosphere.

### Forced expression and down-regulation of NUP37 in HCC cell lines

To generate the NUP37 expression vector, the coding sequence of NUP37 was amplified and inserted into the plasmid pcDNA3.1(containing a myc tag). Transfection was performed using LipofectamineTM 2000 (Invitrogen) according to the manufacturer's instructions. Cells were selected with G418, and the expression of NUP37 was examined using western blot. For knockdown of NUP37 expression, the siRNA lentivirus directed against NUP37 and negative control siRNA were purchased from GeneChem. The virus was incubated with the cancer cells overnight, and cells were selected with puromycine. The NUP37 expression was assessed by Western blot analysis. The expression vectors of HA-TEAD and FLAG-YAP were obtained from Adgene.

### Q-PCR

Quantitative real-time was carried out using SYBR Green Kit (Takara). The sequences of real-time PCR primers were as follows: *NUP37*, forward 5’-GTGAAGATTATGTGCATGTG-3’ and reverse 5’-CTCTAAAACCTTATATTCAT-3’; 18S rRNA, forward 5’-CGGCGACGACCCATTCGAAC-3’ and reverse 5’-GAATCGAACCCTGATTCCCCGTC-3’. The specificity of PCR product was confirmed by melting curve analysis. Each reaction was repeated independently at least three times. The amplification plots were used to determine the threshold cycle (*C*T). The initial mRNA copy numbers for *NUP37* were normalized to that of 18S rRNA.

### Western blot analysis

Whole-cell lysates were prepared with RIPA buffer containing protease inhibitor and phosphatase inhibito. The protein concentrations were measured using Bradford. The following antibodies were used: anti-YAP, anti-CTGF, anti-CYR61 and anti-cyclinE from Cell Signaling Technology; anti-GAPDH from Santa Cruz Biotechnology; and anti-NUP37 from Abcam.

### Luciferase reporter assay

For the luciferase assay, cells were seeded on a 24-well culture plate and grown overnight. The reporter plasmids were co-transfected with the *Renilla* construct (0.04 *μ*g) and *NUP37* siRNA or control siRNA with LipofectamineTM 2000 to assess the transcriptional activity of YAP/TEAD complex. After incubation for 48 hours, the transfected cells were washed twice with PBS, and total proteins were lysed in passive lysis buffer (100 *μ*l/well) provided by the Dual-Luciferase Reporter Assay system (Promega). Firefly and *Renilla* luciferase activities were measured from the lysate using a Centro LB 960 Microplate Luminometer (Berthold). Firefly luciferase values were normalized to *Renilla* luciferase values to control for transfection efficiency.

### Immunofluorescence

Immunofluorescence was completed under standard procedures using a primary antibody against YAP (1:100) or NUP37 (1:1000 dilution). The nucleus was counterstained with DAPI.

### MTT assay

Cells were plated in 96-well plates at the density of 1×10^5^ cells/well. Cell growth was determined using the 3-(4,5-methylthiazol-2-yl)-2,5-diphenyltetrazolium bromide (MTT) colorimetric growth assay for a week. Every other day, cell growth was determined by adding MTT solution (50μg/well) for 4h. Cellular MTT was resolved with DMSO and was measured at 540 nm. All experiments were performed in triplicates.

### Immunohistochemistry

Xylene and ethanol was used to deparaffinized and rehydrated the paraffin-embedded tissue. Endogenous peroxidase activity was blocked with 0.35% H2O2 solution. Antigens retrieve was performed using microwaving. Non-specific binding was blocked by 1% BSA solution Sections were stained with NUP37 antibody and visualized with secondary antibody (Envision, Gene Techenology). Slides were then developed with DAB andcounterstained with hematoxylin.

### Cell migration assay

Boyden chamber was used to evaluate the motility of pancreatic cells. Cells (2×10^5^) suspended in 0.05ml medium containing 1% FBS were placed in the upper chamber, and the lower chamber was loaded with 0.152ml medium containing 10% FBS acting as the chemoattractant. 12 hours later, cells migrated to the lower surface of filters was detected with traditional hematoxylin and eosin (H&E) staining. The experiments were repeated for three times. Five random visual fields were counted for each sample and the average was determined.

### HCC mouse model

The HCC mouse model Alb-Cre; P53^f/f^; Ras^G12D^ was derived by crossing Alb-Cre mice and P53^f/f^; Ras^G12D^ mice. The Alb-Cre mice and P53^f/f^; Ras^G12D^ mice were obtained from Jackson Lab.

### Intrahepatic mouse model

Orthotopic tumors were established by the direct intrahepatic injection of 7404/si con stable cells or 7404/si NUP37 stable cells. Briefly, the left hepatic lobe of each mouse under isoflurane anesthesia was exposed through a small (8-10mm) transverse incision made in the left cranial abdomen beginning 2-3 mm below the xyphoid process and extending to the left. One million 7404/si con or 7404/si NUP37 cells, in a total volume of 0.02 mL of a serum-free medium containing 50% Matrigel (BD Biosciences, Bedford, MA), were slowly injected into the liver through the diaphragmatic surface with a 28-gauge needle. The needle was inserted at a shallow angle so that a gentle injection produced a visible, translucent, subcapsular bleb on the liver surface. After injection, a sterile cotton swab was placed on the needle insertion site as the needle was withdrawn, and gentle pressure was applied for 1 minute to ensure hemostasis. The abdominal musculature and skin were then closed with absorbable suture material and sterile surgical clips, respectively.

### Statistical analysis

Statistical analysis was performed using the SPSS 12.0 software package. Statistical significance was assessed by Student's *t* test. *P*-values of less than 0.05 were considered statistically significant.
